# Oxygenaging: A Physiological Framework for Geroscience

**DOI:** 10.1111/acel.70664

**Published:** 2026-08-28

**Authors:** Stefano Donega, Kenneth W. Fishbein, Paolo Dominelli, Allison B. Herman, Rafael de Cabo, Myriam Gorospe, Luigi Ferrucci

**Affiliations:** ^1^ Translational Gerontology Branch (TGB), National Institute on Aging (NIA), Intramural Research Program (IRP) National Institutes of Health (NIH) Baltimore Maryland USA; ^2^ Laboratory of Clinical Investigation (LCI), NIA, IRP, NIH Baltimore Maryland USA; ^3^ Faculty of Kinesiology University of Calgary Calgary Alberta Canada; ^4^ Laboratory of Cardiovascular Science (LCS), NIA, IRP, NIH Baltimore Maryland USA; ^5^ Laboratory of Genetics and Genomics (LGG), NIA, IRP, NIH Baltimore Maryland USA

**Keywords:** aging, energy, epigenetics, hyperoxia, hypoxia, hypoxia inducible factor 1 (HIF1), mitochondria, oxygen, Oxygenaging, RNA splicing

## Abstract

The stepwise movement of oxygen from the atmosphere to the mitochondria, the “oxygen cascade”, is one of the most tightly regulated systems in physiology. Despite decades of mechanistic study, it has remained quite unexplored in Geroscience. This oversight should be reconsidered. In young organisms, hypoxic stress (whether environmental or tissue‐specific) activates a complex adaptive response to preserve energetic stability via restraining anabolic pathways, optimizing mitochondrial performance, and reinforcing cellular quality control systems. With advancing age, angiostatic signaling increases, endothelial metabolism becomes dysregulated, and overall alveolar ventilation and pulmonary gas exchange (ventilation–perfusion matching and diffusion capacity) become less efficient. These changes promote microvascular rarefaction and low‐grade but persistent mismatches between oxygen delivery and demand at the tissue level, ultimately destabilizing cellular function. In this review, we propose that the gradual erosion of oxygen homeostasis is not simply a byproduct of aging, but also a driver of molecular damage and functional decline. We examine the aging oxygen cascade through the framework of resilience biology, focusing on mechanisms such as mitochondrial electron leaks, oxidative stress amplification, iron dyshomeostasis, ferroptosis, and epigenetic remodeling. We also discuss interventions that alter oxygen availability, such as intermittent hypoxia, hyperbaric oxygen therapy, and hypoxic–hyperoxic training. These approaches demonstrate adaptive potential, but they also highlight the narrow margin between beneficial stress and injury. We propose “Oxygenaging” as a unifying framework in which aging associates with the progressive loss of equilibrium across the oxygen cascade, linking systemic oxygen transport to mitochondrial function, genomic stability, and cellular resilience.

## Graphical abstract

Mechanisms of aging disrupt oxygen homeostasis through three converging processes: oxygen‐cascade impairment, microvascular dysfunction, and molecular maladaptation. “Oxygenaging” integrates these domains to explain how declining oxygen delivery and utilization drive mitochondrial instability and amplify the effect of the hallmarks of aging.

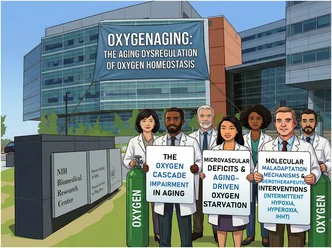

**Abbreviations**
^31^P‐MRS: Phosphorus‐31 Magnetic Resonance Spectroscopy
5hmC: 5‐hydroxymethylcytosine
5mC: 5‐methylcytosine
A–a gradient: alveolar–arterial oxygen difference
ARE: antioxidant response element
ARNT: Aryl Hydrocarbon Receptor Nuclear Translocator (HIF‐1β protein name)
ASL: arterial spin labeling
ATA: atmospheres absolute
ATGL: adipose triglyceride lipase
BDNF: brain‐derived neurotrophic factor
BMAL1: brain and muscle ARNT‐Like 1
BMI: Body Mass Index
BNIP3: BCL2/adenovirus E1B 19 kDa protein‐interacting protein 3
BOLD‐MRI: Blood Oxygen Level–Dependent Magnetic Resonance Imaging
C_a_O_2_
: arterial oxygen content
CD36: cluster of differentiation 36
CLOCK: Circadian Locomotor Output Cycles Kaput
COPD: chronic obstructive pulmonary disease
CPAP: continuous positive airway pressure
CPT1A: Carnitine Palmitoyltransferase 1A
CvO_2_
: venous oxygen content
DLO_2_
: lung diffusion capacity for oxygen
DO_2_
: oxygen delivery
DRP1: dynamin‐related protein 1
ECM: extracellular matrix
eNOS: endothelial nitric oxide synthase
EPCs: endothelial progenitor cells
EPO: erythropoietin
F2‐isoprostanes: markers of lipid peroxidation
FABP: fatty acid‐binding protein
FAO: fatty acid β‐oxidation
FIH‐1: factor inhibiting HIF‐1
F_I_O_2_
: fraction of inspired oxygen
Hb: hemoglobin
HBOT: hyperbaric oxygen therapy
HIF: hypoxia‐inducible factor
HIF‐1α: hypoxia‐inducible factor‐1 alpha
HIF‐1β: hypoxia‐inducible factor‐1 beta (ARNT)
HIF‐2α: hypoxia‐inducible factor‐2 alpha
HIG2: hypoxia‐inducible gene 2
HILPDA: hypoxia‐inducible lipid droplet‐associated protein
hnRNP: heterogeneous nuclear ribonucleoprotein
HPV: hypoxic pulmonary vasoconstriction
HR: heart rate
HSPA9: heat shock protein family A member 9 (mortalin)
HVR: hypoxic ventilatory response
IH: intermittent hypoxia
IHHT: intermittent hypoxic–hyperoxic training
IR: intron retention
JmjC: Jumonji C domain
KDM: lysine‐specific Demethylase
Km: michaelis constant
LCAD: Long‐Chain Acyl‐CoA Dehydrogenase
LIP: labile iron pool
LTF: long‐term facilitation
MCAD: Medium‐Chain Acyl‐CoA Dehydrogenase
MDVs: mitochondrial‐derived vesicles
MIGET: multiple inert gas elimination technique
MRI: magnetic resonance imaging
mtDNA: mitochondrial DNA
mTOR: mechanistic target of rapamycin
MYC: myelocytomatosis oncogene
NAD^+^
: nicotinamide adenine dinucleotide
NCOA4: nuclear receptor coactivator 4
NF‐κB: nuclear factor kappa‐light‐chain‐enhancer of activated B cells
NIRS: near‐infrared spectroscopy
NIX: BNIP3‐like protein
NK cells: natural killer cells
NMD: nonsense‐mediated decay
NO: nitric oxide
NRF2: Nuclear Factor Erythroid 2‐Related Factor 2
OSA: obstructive sleep apnea
OXPHOS: oxidative phosphorylation
p300/CBP: p300/CREB‐Binding Protein
P_a_CO_2_
: arterial carbon dioxide partial pressure
P_A_O_2_
: alveolar oxygen partial pressure
P_a_O_2_
: arterial partial pressure of oxygen
P_B_
: barometric (atmospheric) pressure
PBMC: peripheral blood mononuclear cell
PCE: poison cassette exon
PCr: phosphocreatine
PDH: pyruvate dehydrogenase complex
PDK1: pyruvate dehydrogenase kinase 1
PH_2_O: WATER vapor partial pressure
PHD: prolyl hydroxylase domain
P_is_O_2_
: interstitial oxygen partial pressure
PKM: pyruvate kinase M
PKM1: pyruvate kinase M isoform 1
PKM2: pyruvate kinase M isoform 2
P_mito_O_2_
: mitochondrial oxygen partial pressure
P_mv_O_2_
: microvascular oxygen partial pressure
PPARγ: peroxisome proliferator‐activated receptor gamma
P_t_O_2_
: tissue oxygen partial pressure
Q: cardiac output
Qmax: maximal cardiac output
R: respiratory quotient
R2*: transverse relaxation rate (used in BOLD‐MRI)
REDD1: Regulated in Development and DNA Damage Responses 1
REP: renal erythropoietin‐producing
ROS: reactive oxygen species
rScO_2_
: regional cerebral oxygen saturation
S_a_O_2_
: arterial oxygen saturation
SASP: senescence‐associated secretory phenotype
SDH: succinate dehydrogenase
SDQ: log standard deviation of perfusion distribution
SIRT1: Sirtuin 1
S_m_O_2_
: skeletal muscle oxygen saturation
SP1: transcription factor
SRSF: serine/arginine‐rich splicing factor
SV: stroke volume
TCA: tricarboxylic acid
TcPO_2_
: transcutaneous oxygen partial pressure
TET: ten‐eleven translocation dioxygenases
TFAM: mitochondrial transcription factor A
TrkB: tropomyosin receptor kinase B
TSP‐1: thrombospondin‐1
UCP3: uncoupling protein 3
V/Q: ventilation–perfusion ratio
VEGF: vascular endothelial growth factor
VEGFR‐2: vascular endothelial growth factor receptor 2
VHL: Von Hippel–Lindau Tumor Suppressor
VO_2_max: maximal oxygen uptake
α‐KG: alpha‐ketoglutarate
τPCr: Phosphocreatine recovery time constant

## 1 The Oxygen Cascade Impairment in Aging

The rise in atmospheric oxygen was a key factor enabling the evolution of complex multicellular life. Because of its essential role in energy production as the terminal electron acceptor in oxidative phosphorylation (OxPhos), a complex ensemble of coordinated physiological and molecular systems was evolutionarily selected to optimize uptake of oxygen from the air, supply it to tissues through the vascular system, and deliver it to mitochondria in tissues and organs (Box 1). The systems comprising this ensemble are intertwined at multiple levels and bidirectionally (Pittman 2011; Hepple 2016) with established genomic and metabolic hallmarks of aging (Lopez‐Otin et al. 2013; Lopez‐Otin et al. 2023). An extensive review of the literature, although considerably fragmented, reveals that dysfunction at multiple levels of oxygen delivery is a main cause of aging. We invite the reader on a journey from the atmosphere to the mitochondrial matrix. Step by step, we follow the drop in oxygen partial pressure across the cascade, and argue that breakdowns in macroscopic transport systems reverberate downward, destabilizing molecular compensation, triggering canonical hallmarks of aging, and accelerating tissue dysfunction. To highlight the multifactorial age‐related erosion of cellular resilience to changes in available oxygen, we propose here the term Oxygenaging as a framework that links systemic physiology to Geroscience.

**FIGURE 1:**
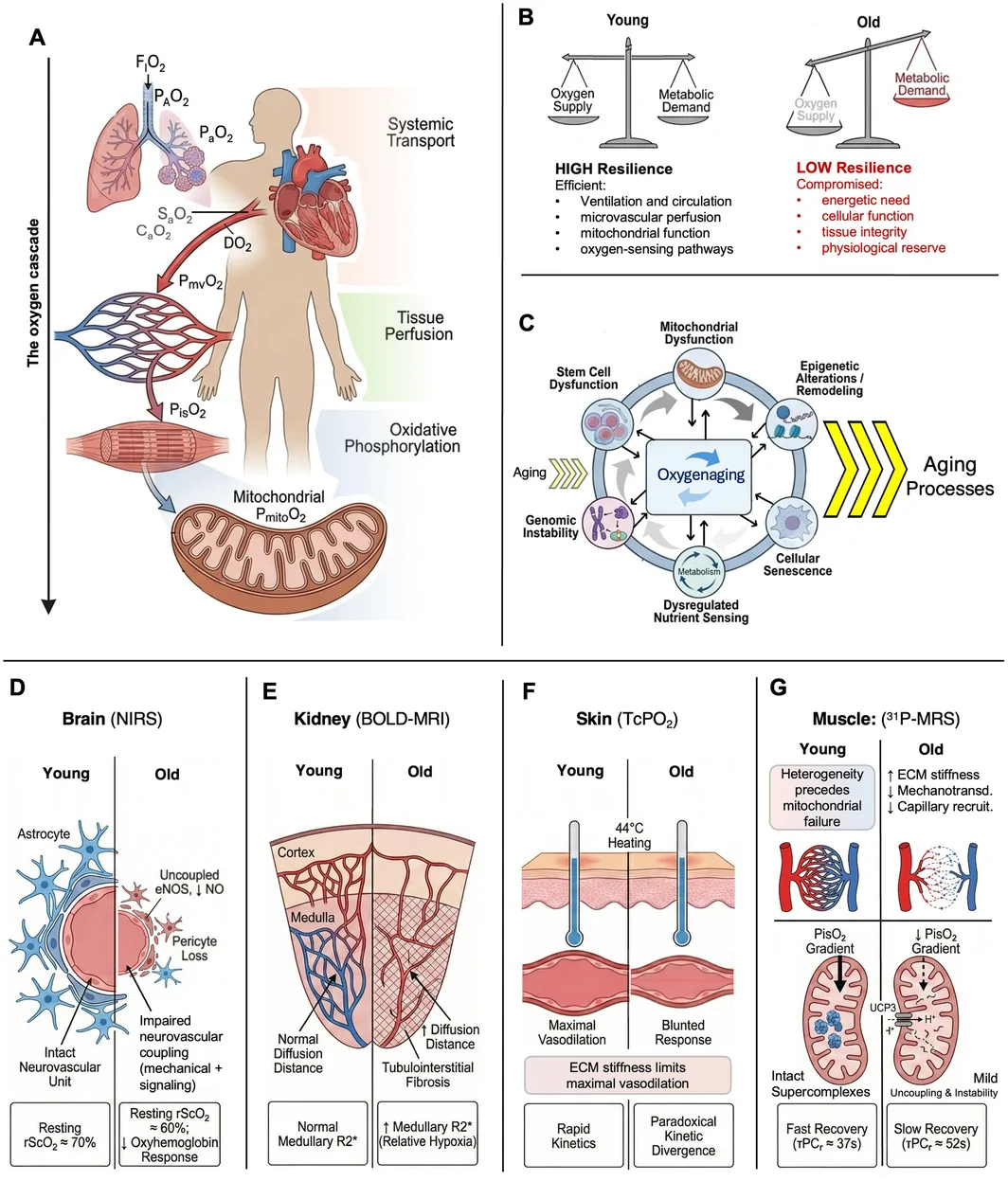
Oxygenaging: A Systems‐Level Physiological Framework and Organ‐Specific Manifestations. (A) The Multiscale Oxygen Cascade. Sequential drop in oxygen partial pressure across biological scales, tracking transport from macroscopic pulmonary and cardiovascular delivery to microscopic tissue perfusion and mitochondrial oxidative phosphorylation. (B) Progressive Loss of Homeostatic Balance. Young organisms dynamically maintain high resilience by matching oxygen supply to metabolic demand. Older organisms exhibit a loss of resilience and supply–demand mismatch (further exacerbated by environmental modifiers such as physical inactivity and obesity, not displayed in the figure). (C) Hallmarks of Aging Involved in Oxygenaging speeding up aging processes. Bidirectional schematic interactions between the progressive loss of oxygen homeostasis and 6 out of 12 known and established hallmarks of aging. (D) Brain (NIRS). Schematic of the neurovascular unit comparing intact pericytes and robust coupling in youth versus the aged state, characterized by pericyte degeneration, eNOS uncoupling, and Blood–Brain Barrier leakage, leading to reduced regional cerebral oxygen saturation. (E) Kidney (BOLD‐MRI). Map of the corticomedullary oxygen gradient. In the aged kidney, peritubular capillary rarefaction and tubulointerstitial fibrosis enlarge diffusion distances, causing relative medullary hypoxia. (F) Skin (TcPO_2_). The skin from older individuals shows a blunted vasodilatory response to maximal thermal stress (44°C) due to increased extracellular matrix (ECM) stiffness, limiting maximal vessel expansion. (G) Muscle (^31^P‐MRS). Comparison of mitochondrial efficiency and microvascular refilling. Aged muscle shows slower phosphocreatine recovery kinetics (PCr 52 s vs. 37 s in youth), paralleled by mild uncoupling by UCP3 and respiratory supercomplex instability. F_I_O_2_: Fraction of inspired oxygen; P_A_O_2_: Alveolar oxygen partial pressure; P_a_O_2_: Arterial oxygen partial pressure; S_a_O_2_: Arterial oxygen saturation (percentage of hemoglobin binding sites that are occupied by oxygen in the arterial blood); CaO_2_: Arterial oxygen content; DO_2_: Systemic oxygen delivery; P_mv_O_2_: Microvascular oxygen partial pressure; P_is_O_2_: Interstitial oxygen partial pressure; P_mito_O_2_: Mitochondrial oxygen partial pressure; rScO2: Regional cerebral oxygen saturation; R2*: Transverse relaxation rate (surrogate marker of tissue hypoxia); TcPO_2_: Transcutaneous oxygen partial pressure; ^31^P‐MRS: Phosphorus‐31 Magnetic Resonance Spectroscopy; PCr: Phosphocreatine recovery time constant.

The oxygen cascade begins when oxygen is inhaled from the fraction of inspired oxygen (F_I_O_2_) in the atmosphere and transported via convection through the bronchial tree to the alveoli. There, oxygen generates an alveolar partial pressure (P_A_O_2_) that exceeds that of venous blood, creating the gradient that drives diffusion across the alveolar–capillary membrane and raises arterial oxygen partial pressure (P_a_O_2_). Once in blood, oxygen binds to hemoglobin, as characterized by arterial oxygen saturation (S_a_O_2_, the percentage of hemoglobin binding sites that are occupied by oxygen in the arterial blood). Hemoglobin‐bound oxygen accounts for most total arterial oxygen content (C_a_O_2_), alongside a minor dissolved fraction. Oxygen is transported to the tissues by convective blood flow driven by cardiac output (Q), with oxygen delivery (DO_2_ = Q × CaO_2_) reflecting the amount of oxygen carried to the microcirculation, where a progressive decline in partial pressure from the microvascular compartment (P_mv_O_2_) to the interstitium (P_is_O_2_) drives diffusion into cells and ultimately into the mitochondrial matrix (P_mito_O_2_) where oxidative phosphorylation occurs. At the intracellular level, oxygen availability is further influenced by oxygen‐binding globins such as myoglobin in muscle and neuroglobin in neural tissues. Myoglobin facilitates intracellular oxygen diffusion and buffers fluctuations in oxygen supply, whereas neuroglobin is thought to support cellular adaptation and resilience under hypoxic or ischemic stress (Wittenberg and Wittenberg 2003; Burmester et al. 2000). Age‐related alterations in these systems remain incompletely understood but may represent additional components of oxygen homeostasis. The cascade can be summarized below and visually in Figure 1A:

$$\begin{array}{c} F_{I}O_{2}\to P_{A}O_{2}\to P_{a}O_{2}\to \left(S_{a}O_{2}\&C_{a}O_{2}\right)\\ \;\to {\mathrm{DO}}_{2}\to P_{\mathrm{mv}}O_{2}\to P_{\text{is}}O_{2}\to P_{\text{mito}}O_{2}. \end{array}$$

The efficiency of the oxygen cascade is strongly influenced by lifestyle and behavioral factors. Regular physical activity enhances ventilatory function, cardiovascular performance, microvascular density, endothelial health, and mitochondrial oxidative capacity, whereas chronic inactivity has opposite effects (Hawley et al. 2014). Similarly, obesity can impair oxygen homeostasis through altered pulmonary mechanics, systemic inflammation, endothelial dysfunction, and reduced metabolic flexibility (Van Gaal et al. 2006; Dixon and Peters 2018). Therefore, Oxygenaging should not be viewed as a process determined solely by chronological aging, but rather as the result of interactions between biological aging and lifelong environmental exposures. Many hallmarks of aging, including mitochondrial dysfunction, inflammation, extracellular matrix remodeling, and cellular senescence, can impair oxygen transport and utilization. Conversely, disturbances in oxygen homeostasis may amplify these same processes. We therefore propose Oxygenaging as a physiological framework that integrates bidirectional interactions among molecular hallmarks, tissue physiology, and organismal resilience rather than as a singular causal pathway.

A central concept of the Oxygenaging framework is that physiological function depends not on absolute oxygen availability alone, but on the capacity to continuously match oxygen supply and metabolic demand. Young organisms maintain this balance through highly coordinated adaptations involving ventilation, circulation, microvascular perfusion, mitochondrial function, and oxygen‐sensing pathways. Consequently, Oxygenaging should not be interpreted as a state of oxygen deficiency per se. Rather, it reflects a progressive loss of resilience in the mechanisms that maintain oxygen homeostasis across changing physiological conditions. As this adaptive capacity erodes, organisms become increasingly vulnerable to mismatches between oxygen availability and energetic requirements, compromising cellular function, tissue integrity, and physiological reserve (Figure 1B). Impairment of oxygen homeostasis may both contribute to and result from established hallmarks of aging, creating maladaptive feedback loops that amplify molecular damage, reduce physiological reserve, and accelerate functional decline.

In this review, we propose Oxygenaging as a physiological framework linking systemic oxygen transport, tissue oxygen availability, mitochondrial function, and molecular hallmarks of aging. Rather than representing a single causal mechanism, Oxygenaging highlights how progressive loss of resilience across the oxygen cascade may interact with and amplify established aging processes, ultimately contributing to functional decline and loss of physiological reserve. Oxygenaging should not be viewed as an alternative to the Hallmarks of Aging framework. Rather, we propose that oxygen homeostasis represents a physiological axis that intersects with multiple hallmarks of aging. Impaired oxygen delivery and utilization can contribute to mitochondrial dysfunction, chronic inflammation, epigenetic remodeling, altered nutrient sensing, stem cell dysfunction, and cellular senescence. Conversely, these same hallmarks may impair oxygen transport, oxygen sensing, and mitochondrial oxygen utilization. Thus, Oxygenaging may represent a physiological interface through which molecular hallmarks interact and amplify one another (Figure 1C).

BOX 1 **Evolutionary Framing of Oxygenaging.**

It is well established that the most significant event in cell evolution was mitochondrial endosymbiosis, which drastically increased ATP availability and enabled the development of large genomes and complex tissues (Gray et al. 1999). In fact, “cellular life” developed from an anoxic world to one with significant oxygen levels, through the Proterozoic eon (2500–538 million years ago), during which atmospheric and oceanic oxygen rose in a stepwise manner and progressively reshaped Earth's redox landscape (Holland 2006). These changing oxygen conditions set the stage for the emergence of early Metazoans, originating between 750 and 800 million years ago (Lyons et al. 2014). Cells subsequently evolved conserved oxygen‐sensing systems, including the HIF pathway, which coordinates angiogenesis, metabolism, and survival during hypoxia and is optimized for acute fluctuations in oxygen supply rather than long‐term maintenance across decades of life (Semenza 2012). The presence of the HIF system across metazoans further reflects its ancient evolutionary origin (Rytkonen and Storz 2011). In this temporal frame, the primary ATP‐generating systems were based on oxidative phosphorylation, exposing cells to the intrinsic chemical reactivity of O_2_ and the formation of reactive oxygen species (Imlay 2003). Comparative genomic analyses show that (I) terminal oxidases and dioxygen reductases were shaped by horizontal gene transfer among early ancestors (the movement of genetic material among microbial organisms other than by parent to offspring‐vertical transmission) (Brochier‐Armanet et al. 2009), and that (II) mitochondrial respiratory O_2_/NO reductase families display complex and non‐linear evolutionary trajectories (Ducluzeau et al. 2014). Since life‐history theory shows that animals must allocate finite energetic resources among growth, reproduction, and somatic maintenance, aging can be viewed as the late‐life destabilization of oxygen‐handling systems (Kirkwood 1977). In this context, aging reveals intrinsic vulnerabilities of oxygen‐based metabolism rather than failures of systems that were never optimized for longevity (Speakman and Selman 2011).

## 2 The First Oxygenaging Step: Pulmonary Gas Exchange

The oxygen cascade entry point is the alveolar oxygen pressure (P_A_O_2_). P_A_O_2_ is calculated using the simplified alveolar gas equation (Stickland et al. 2013), and expanded below to include standard variables:

$$P_{A}O_{2}=\left[\left(P_{B}-{\mathrm{PH}}_{2}O\right)\times F_{I}O_{2}\right]–\left(P_{a}{\mathrm{CO}}_{2}/R\right).$$

From the equation, F_I_O_2_ is the fraction of inspired oxygen (~0.21) and P_B_ is the atmospheric pressure (~760 mmHg at sea level); PH_2_O is the standard water vapor pressure at body temperature (~47 mmHg); P_a_CO_2_ is the arterial carbon dioxide partial pressure, which reflects alveolar ventilation as CO_2_ displaces oxygen in the alveoli (~40 mmHg, used by convention as a surrogate for alveolar CO_2_, though minor alveolar‐arterial CO_2_ gradients may emerge in older populations); and R is the ratio of CO_2_ produced to O_2_ consumed (respiratory quotient; ~0.8) (Fenn et al. 1946; West and Luks 2016). While highly variable among individuals of the same age, the age‐dependent structural integrity of the lungs (Bachofen et al. 1973) governs the efficiency of the actual transition from alveolar air to arterial blood, which is measured by the alveolar‐arterial oxygen gradient P (A − a) O_2_.

Age‐related remodeling of the lung parenchyma and the intrinsic loss of elastin fibers reduce lung elastic recoil (Subramaniam et al. 2017). The resulting decrease in tethering forces on small airways promotes premature airway closure, particularly in gravity‐dependent regions of the lung (Janssens et al. 1999; Sharma and Goodwin 2006). This loss of elastic recoil further impairs oxygen equilibration, widening the normal gap between alveolar and arterial oxygen partial pressures. These poorly ventilated but still perfused units constitute regions with low ventilation‐to‐perfusion ratios (V/Q) that behave as functional shunts, preventing full equilibration between alveolar and arterial oxygen and widening the P (A − a) O_2_ gradient, as dynamic impairment of gas exchange. Furthermore, aging introduces important mechanical limits to ventilation: while often clinically silent at rest, this loss of ventilatory reserve restricts the ability to increase P_A_O_2_ during exercise or physical stress (Johnson et al. 1991). Aging severely exacerbates this preexisting physiological heterogeneity: in pulmonary arterial oxygenation, this spatial heterogeneity is quantified by the log standard deviation of the perfusion distribution (SDQ), an index used to quantify V/Q heterogeneity in Multiple Inert Gas Elimination Technique (MIGET) studies. In the aging lung, the loss of elastic recoil leads to premature airway closure and an increase in this V/Q heterogeneity. The SDQ rises significantly from ~0.36 at younger ages to ~0.47 at older ages (Cardus et al. 1997). This is a notable shift toward dysfunction, though still well below the severe heterogeneity seen in chronic obstructive pulmonary disease (COPD), where SDQ often exceeds ~0.8. Early predictive models (Cerveri et al. 1995) established the reference equation for the arterial oxygen partial pressure:

$$P_{a}O_{2}=143.6-\left(0.39\times \mathrm{Age}\right)-\left(0.56\times \mathrm{BMI}\right)-\left(0.57\times P_{a}{\mathrm{CO}}_{2}\right).$$

This empirical relationship indicates that age is the most robust universal predictor of arterial oxygenation, resulting in a P_a_O_2_ drop of roughly 15–20 mmHg between mid‐life and older age. In addition to age, higher body mass index (BMI) and P_a_CO_2_ are independently associated with lower arterial oxygen tension, likely reflecting mechanical and ventilation‐to‐perfusion constraints with alveolar hypoventilation.

Despite early impairment in pulmonary oxygen transfer, resting arterial oxygenation is usually preserved, as the flat upper plateau of the oxygen‐hemoglobin dissociation curve allows hemoglobin to remain nearly fully saturated over a wide range of PO_2_. Notably, the intersection of oxygen sensing and iron homeostasis impacts these earliest steps of the cascade–ultimately driving the mitochondrial toxicity (discussed later in this review).

Hypoxic Pulmonary Vasoconstriction (HPV) is an autoregulatory mechanism meant to divert blood away from poorly ventilated alveoli to optimize V/Q matching. However, HPV can be pathologically exacerbated by systemic iron deficiency (a common aging feature), which mimics hypoxia at the level of the pulmonary endothelium by inhibiting Prolyl Hydroxylase Domain (PHD) enzymes (Frise et al. 2016; Frise and Robbins 2015). In older adults, this exaggerated HPV response worsens overall spatial V/Q mismatch and elevates pulmonary vascular resistance, placing additional mechanical strain on the right ventricle and further threatening the central cardiac output (Q) required for the next step of the cascade (Cotroneo et al. 2015; Hartmann et al. 2023).

## 3 The Second Oxygenaging Step: Central Hemodynamics

The oxygen available to tissues is determined by arterial oxygen content (C_a_O_2_), which depends mainly on hemoglobin concentration [Hb] and S_a_O_2_ because the quantity of free dissolved oxygen is a minor component. The relationship is summarized by the equation (Treacher and Leach 1998):

$$C_{a}O_{2}=\left(1.34\times \left[\mathrm{Hb}\right]\times S_{a}O_{2}\right)+\left(0.003\times P_{a}O_{2}\right)$$

The sigmoidal shape of the hemoglobin–oxygen dissociation curve influences how pulmonary structural changes translate into arterial oxygenation. The P_a_O_2_ at which [Hb] is 50% saturated (P_50_ of the dissociation curve, typically ~27 mmHg) (Kaminsky et al. 2013) does not undergo a compensatory rightward shift to facilitate tissue unloading in older adults. While individual erythrocytes experience a leftward shift (increased oxygen affinity) as they circulate and deplete their 2,3‐bisphosphoglycerate (2,3‐BPG) stores over their ~120‐day cellular lifespan (Samaja et al. 1990), healthy chronological human aging does not spontaneously elevate baseline systemic 2,3‐BPG levels to force a compensatory rightward shift (Humpeler et al. 1989). Because the aging cardiovascular system lacks a baseline rightward shift to proactively facilitate oxygen unloading at the tissue level, systemic oxygen delivery becomes highly dependent on local metabolic triggers.

In the aging lung, poorly ventilated low V/Q units behave as functional shunts delivering partially deoxygenated blood (e.g., with an oxygen saturation of ~75%) into the pulmonary venous circulation, while healthy units cannot passively compensate via overventilation to provide highly oxygenated blood (e.g., ~98%). However, because the oxyhemoglobin dissociation curve has a sigmoid shape with a flat upper plateau, overventilation in these high V/Q regions produces only minimal additional increases in hemoglobin saturation (West and Luks 2016). In contrast, low regions operate on the steep portion of the curve, where small reductions in P_a_O_2_ cause substantial drops in saturation. The mixing of these distinct blood volumes leads to a significantly lower arterial saturation in the aged lung due to the combined deficits of increased shunting in the hypoventilated areas and lack of compensation in the overventilated areas. In old individuals with arterial oxygenation (baseline P_a_O_2_) already compromised by the mismatching, this stability in the standard curve weakens the relationship between P_A_O_2_ and P_a_O_2_ (West and Luks 2016). Furthermore, because the baseline curve is not pre‐adapted for enhanced unloading, the system relies heavily on acute stressors (e.g., the concurrent increases in core temperature and local acidity during exercise, also called the Bohr effect) to drive the curve to the right and prevent a severe tissue loading deficit. While models of this decoupling in healthy aging are scarce, a similarly severe impairment in gas exchange (decoupling) has been described in the profound mismatches that occur in severe COVID‐19, providing a striking pathological analogue to the gradual decline seen in the aging lung (Ceruti et al. 2022).

The critical variable for tissue survival is oxygen delivery (DO_2_) that is defined as the product of C_a_O_2_ and cardiac output (Q; reflecting global macro‐circulation rather than local microvascular perfusion) (Treacher and Leach 1998):

$${\mathrm{DO}}_{2}=C_{a}O_{2}\times Q$$

In this context, Q is the product of heart rate (HR) and stroke volume (SV). HR reflects chronotropic activity and SV represents the volume of blood ejected by the left ventricle into the arterial system per beat. Oxygen delivery is therefore:

$${\mathrm{DO}}_{2}=C_{a}O_{2}\times \left(\mathrm{HR}\times \mathrm{SV}\right)$$

Since DO_2_ depends on both blood flow and C_a_O_2_, in aging the decline in Q (particularly in HR), becomes the dominant limitation on peak oxygen delivery. The deficit is then compounded by factors that can lower C_a_O_2_, such as a decline in S_a_O_2_, although [Hb] itself remains stable as long as erythropoiesis is preserved. For context, the relative magnitude of this structural cardiovascular decline is unambiguous: HR may drop by roughly 25% (e.g., from ~200 bpm in youth to ~150 bpm in older age), whereas C_a_O_2_ experiences a much smaller relative reduction.

## 4 The Third Oxygenaging Step: Microvascular Diffusion

The integrated performance of the oxygen cascade from ventilation and pulmonary diffusion to cardiovascular delivery and peripheral oxygen extraction is called maximal oxygen uptake (VO_2_max) and is calculated using the Fick equation:

$${\mathrm{VO}}_{\text{2max}}=Q_{\max}\times \left(C_{a}O_{2}-C_{v}O_{2}\right)$$

In this equation, C_v_O_2_ is the oxygen content in venous blood under maximum exertion. C_a_O_2_ is only modestly reduced by age. As a consequence, VO_2_max is sharply limited by two concurrent processes: a central reduction in maximal heart rate (lowering Qmax) and a peripheral failure in oxygen extraction. As a result of the upstream deficits previously described, maximal oxygen uptake (VO_2_max) falls by ~10% per decade, a decay that fundamentally reduces overall cardiovascular functional capacity (Betik and Hepple 2008). To understand this peripheral failure, the focus must shift to the microvasculature, as this step is critically impaired in Oxygenaging. Aging is associated with progressive dysfunction of the signaling networks that regulate capillary perfusion to match oxygen and nutrient delivery to local metabolic demand. Impairment of endothelial, neurohumoral, and metabolic vasodilatory pathways reduces the precision of microvascular flow control. In parallel, chronic low‐grade inflammation (characterized by interstitial edema, immune cell infiltration, and fibrosis) further disrupts capillary exchange dynamics. Thus, even if the structural vascular networks are preserved, with apparently adequate bulk blood flow, older individuals may exhibit impaired vasomotor regulation, leading to perfusion heterogeneity and microregional hypoxia.

Importantly, the Fick equation previously displayed only describes the total volume of oxygen removed from the circulation, without capturing the local heterogeneity of oxygen transfer that emerges at the microvascular interface (Haseler et al. 2007). In a steady physiological state, the rate of oxygen extraction from the bulk flow (VO_2_) equals the rate of oxygen diffusion from the microvasculature into the cells. At the tissue level, oxygen transfer is governed not only by convective delivery but also by diffusion from the microvasculature to the mitochondria. This process follows a diffusion‐based equation of Fick's law (Wagner 1996), commonly expressed in the context of skeletal muscle physiology, as follows:

$${\mathrm{VO}}_{2}={\mathrm{DLO}}_{2}\times \left(P_{\mathrm{mv}}O_{2}-P_{\text{mito}}O_{2}\right)$$

In this equation, tissue oxygen consumption (VO_2_) is determined by the effective oxygen diffusion capacity (DLO_2_) multiplied by the partial pressure gradient between the microvasculature oxygen partial tension (P_mv_O_2_) and the mitochondria oxygen tension (P_mito_O_2_) (Barstow 2019). Aging is characterized by reduced DLO_2_ through microvascular rarefaction, endothelial dysfunction (including reduced eNOS/NO bioavailability), and increased diffusion distances caused by fibrosis and diminished pericyte coverage across organs such as the brain, kidney, skin, and muscle (Hepple 2016; Ungvari, Tarantini, Donato, et al. 2018). These limitations are severely exacerbated during exercise hyperemia: while healthy young tissue rapidly matches metabolic demand through swift capillary recruitment and vasodilation, aged microvasculature exhibits a “slow” hyperemic response. This blunted vascular reactivity is driven by impaired sympathetic withdrawal and compromised local metabolic signaling and restricts the necessary rise in P_mv_O_2_, strictly limiting the diffusion gradient at the exact moment it is needed most. As a result of this local diffusion failure, even when overall Q and C_a_O_2_ remain preserved, VO_2_ precipitously declines. This localized diffusion failure narrows the effective oxygen pressure gradient across the interstitium, thereby reducing the driving force for mitochondrial oxygen uptake. Although skeletal muscle is used here as the primary bioenergetic model (owing to the availability of robust non‐invasive measurements), comparable age‐related mitochondrial declines have been documented across multiple tissues (Betik and Hepple 2008).

During active aging muscle contraction, the extracellular oxygen tension provided by the microvasculature, the interstitial oxygen pressure (P_is_O_2_), falls lower in older than in young muscle (Pittman 2011; Behnke et al. 2005). This drop indirectly constrains and compromises the last step of oxidative phosphorylation, with impairment in electron transfer efficiency and increased electron leak (Genova and Lenaz 2015). This leak not only blunts ATP production efficiency, but it also acts as the primary source of the chronic reactive oxygen species (ROS) production that fuels the “pseudohypoxic” molecular signaling discussed in the following sections (Amara et al. 2007; Balsa et al. 2019). Table 1 summarizes the three Oxygenaging steps discussed.

**TABLE 1:** The “Oxygenaging” cascade: Sequential impairments in oxygen transport.

Step	Component	Variable	Biological and functional outcome	References
Pulmonary gas exchange	Lung parenchyma mechanics	P_A_O_2_	Intrinsic loss of elastin fibers leads to premature airway closure in gravity‐dependent lung regions. It limits inhaled oxygen entering the alveoli	(Subramaniam et al. 2017; Janssens et al. 1999; Sharma and Goodwin 2006)
		P_a_O_2_		
	Ventilation‐perfusion (V/Q) matching	↑ P_(A‐a)_O_2_ gradient	Age‐related formation of poorly ventilated functional shunts causes a measurable fall (typically 15–20 mm Hg) in resting arterial oxygenation	(Cardus et al. 1997; Cerveri et al. 1995)
		↑ SDQ		
		↓ P_a_O_2_		
	Ventilatory reserve	P_A_O_2_ (during stress)	Aging progressively diminishes the mechanical efficiency of the respiratory system. While this rarely affects resting breathing, it makes increasing oxygen intake during physical activity substantially harder	(Johnson et al. 1991)
Central hemo‐dynamics	Hemoglobin‐oxygen affinity	Stable P_50_	Failure to raise baseline systemic 2,3‐BPG prevents the hemoglobin–oxygen curve from shifting rightward, forcing tissues to rely solely on exercise‐induced heat and acidosis (the Bohr effect) for oxygen unloading	(Kaminsky et al. 2013; Samaja et al. 1990; Humpeler et al. 1989; Ceruti et al. 2022)
	Pulmonary venous admixture	↓ SaO_2_	The flat plateau of the oxygen‐hemoglobin curve prevents healthy alveoli from over‐compensating for failing units, dropping overall arterial saturation (e.g., from ~92% to ~85%).	(West and Luks 2016)
	Cardiac output (macro‐circulation)	↓ HRmax	Age‐related declines in chronotropic responsiveness (producing ~25% lower maximal heart rates) and reduced stroke‐volume reserve independently constrain maximal cardiac output (Q̇max), forming the primary bottleneck to bulk oxygen delivery.	(Treacher and Leach 1998)
		↓ Qmax		
		↓ DO_2_		
Micro‐vascular diffusion	Vasomotor regulation	↓ PmvO_2_	Breakdown of endothelial signaling and impaired sympathetic withdrawal produce a sluggish hyperemic response, depriving muscle of the rapid oxygen surge normally delivered at the onset of exertion	(Haseler et al. 2007; Barstow 2019)
	Microvascular structure	↓ DLO_2_	Microvascular rarefaction, fibrosis, and interstitial edema widen diffusion distances, slowing oxygen transfer and contributing to an approximate 10%‐per‐decade decline in VO_2_max	(Hepple 2016; Betik and Hepple 2008; Ungvari, Tarantini, Donato, et al. 2018)
		↓ VO_2_max		
	Mitochondrial oxidative phosphorylation	↓ P_is_O_2_	Reduced oxygen availability compromises respiratory complexes: cells produce less energy (ATP) and leak harmful free radicals (ROS), driving chronic “pseudohypoxic” damage	(Pittman 2011; Behnke et al. 2005; Genova and Lenaz 2015; Amara et al. 2007; Balsa et al. 2019)
		↓ P_mito_O_2_		
		↑ ROS		

## 5 Microvascular Deficits and Aging‐Driven Oxygen Starvation

The structural and functional microvascular failure reflects a deep breakdown in the metabolic machinery required for effective angiogenesis. Specifically, an underappreciated dichotomy lies in the metabolic specialization of endothelial cells compared to parenchymal cells. Endothelial cell energetics primarily relies on glycolysis rather than oxidative phosphorylation, enabling angiogenesis even in low‐oxygen environments (Leung and Shi 2022; Du 2021; Dalal et al. 2025). Aging disrupts this metabolic specialization through impaired glycolysis, mitochondrial dysfunction, and altered redox signaling, collectively reducing endothelial migratory, proliferative, and barrier‐remodeling capacity required for angiogenesis (Ungvari, Tarantini, Kiss, et al. 2018). Even if endothelial cells are capable of sensing oxygen, the machinery to carry out angiogenesis may no longer function due to metabolic dysfunction (Wang et al. 2025). As a result, endothelial metabolic failure presents a unique bottleneck in the oxygen cascade and provides a rationale for why oxygen supplementation or increased availability may be unsuccessful at rescuing microvascular function with age. Although the mechanisms discussed here are presented as common components of the Oxygenaging framework, their relative contribution is likely to differ across tissues and organs. Aging does not occur synchronously throughout the body. Instead, organ systems follow partially independent trajectories shaped by distinct metabolic demands, regenerative capacities, vascular architectures, and environmental exposures. Consequently, Oxygenaging should be viewed as a systems‐level framework within which tissue‐specific manifestations emerge through different combinations of impaired oxygen delivery, altered oxygen sensing, mitochondrial dysfunction, and adaptive failure.

Below, we present a few organ/tissue‐specific contexts.

### 5.1 Brain

Regional cerebral oxygen saturation (rScO_2_) can be estimated by near‐infrared spectroscopy (NIRS, Figure 1D). However, since NIRS detects a composite signal of arterial and venous blood (~25:75 ratio) and includes scalp contributions, rScO_2_ cannot be considered equivalent to brain arterial saturation (S_a_O_2_). A systematic review confirmed that resting frontal rScO_2_ declines with age (Yeung and Chan 2021). Age‐stratified measurements show that rScO_2_ decreases from ~69.8% in young adults (mean age 26) to ~62.7% in older adults (Lian et al. 2020). A large prospective study (*n* = 1616) confirmed that resting rScO_2_ declines across age groups: median values dropped from 67% (18–49 years) to 63% (50–74 years) and 60% (≥ 75 years), with notably lower baselines in women (Robu et al. 2020). Blood Oxygenation Level‐Dependent magnetic resonance imaging (BOLD‐MRI) allowed whole‐brain oxygenation mapping without scalp interference by measuring a signal sensitive to the concentration of deoxyhemoglobin, which increases the proton R_2_* relaxation rate (Ogawa et al. 1990). While BOLD‐MRI provides spatially resolved oxygenation maps, NIRS remains valuable for capturing rapid dynamic changes due to its temporal resolution and accessibility. In healthy older adults during active stand, NIRS detected a 4.6 μM drop in frontal oxyhemoglobin, indicating a blunted oxyhemoglobin response to stress that is not observed in young adults (Mehagnoul‐Schipper et al. 2000). This largely reflects endothelial senescence, microvascular rarefaction, and decreased nitric oxide availability (Yang et al. 2009). This vascular aging phenotype was attributed to oxidative stress and inflammation that impair neurovascular coupling (Ungvari, Tarantini, Donato, et al. 2018). Specifically, the uncoupling of endothelial nitric oxide synthase (eNOS) reduces the bioavailability of nitric oxide (NO), which impairs the quick cerebrovascular reactivity required to maintain perfusion pressure during challenges, such as orthostatic stress. Importantly, while absolute cerebral blood flow progressively drops with age, directly constraining bulk systemic oxygen delivery (DO_2_, described in the previous sections), the fundamental mechanisms of cerebral autoregulation generally remain intact in healthy older adults (Weijs et al. 2024). The deficit is not necessarily a failure of autoregulation itself, but rather a structurally lower baseline flow compounded by sluggish reactivity. Together with capillary stiffening, which impairs the physical ability of the vessel to dilate and disrupts biochemical cross‐talk from neurons/astrocytes, this neurovascular decoupling leads to the reduced resting rScO_2_ (Ungvari, Tarantini, Donato, et al. 2018). The neurovascular decoupling is also driven by the age‐dependent degeneration of pericytes, which are essential for maintaining blood–brain barrier integrity and regulating capillary diameter (Tarantini et al. 2017; Montagne et al. 2015). This breakdown of the neurovascular unit is a central feature of cognitive aging and neurodegeneration (Iadecola 2017).

### 5.2 Kidney

Renal BOLD‐MRI infers relative deoxyhemoglobin concentration via the transverse relaxation rate R_2_* (Figure 1E). While cortical oxygenation remains relatively preserved with aging, medullary R_2_* values (a surrogate marker of tissue hypoxia) increase progressively, consistent with worsening medullary hypoxia (Li, Yang, et al. 2019). The resulting amplification of the corticomedullary oxygen gradient likely arises from peritubular capillary rarefaction and tubulointerstitial fibrosis, which lengthen diffusion distances and reduce effective oxygen delivery (Tanaka and Nangaku 2013).

### 5.3 Skin

Transcutaneous oxygen partial pressure (TcPO_2_; Figure 1F) estimates near‐surface capillary oxygen tension (Wimberley et al. 1985), although values depend heavily on probe temperature. A paradoxical divergence in vasomotor kinetics was observed: TcPO_2_ decreased with age when the probe was heated to 44°C (a temperature that activates C fiber‐mediated neurogenic vasodilation) but increased relative to young controls at 39°C (Ogrin et al. 2005). When measurements are interpreted at the same probe temperature, the apparent paradox disappears, because the age effect reflects a reduced ability to mount sensory nerve‐driven vasodilation rather than an actual vasoconstrictive response. This blunted response suggests that extracellular matrix (ECM) stiffness mechanically limits maximal vessel expansion (Holowatz et al. 2003; Holowatz et al. 2008), and it is also consistent with age‐related declines in C‐fiber function that impair neurogenic inflammatory vasodilation. Meanwhile, the finding at lower temperatures may reflect altered skin diffusivity or metabolic demand rather than superior vasodilation and may also be influenced by reduced sympathetic vasoconstrictor tone in older adults, which increases basal perfusion and thereby elevates TcPO_2_ when the skin is warmed, but not enough to activate sensory nerves.

### 5.4 Muscle

NIRS has been used to explore local microvascular hemoglobin saturation in skeletal muscle under different conditions (S_m_O_2_; Figure 1G). While saturation is not equivalent to oxygen partial pressure, S_m_O_2_ closely reflects the capillary oxygen tension that determines the gradient for diffusion into the interstitium (P_is_O_2_). Using this method, older adults were found to reoxygenate significantly slower post‐exercise compared to rapid recovery in young controls (Chung et al. 2018). Impaired interstitium‐to‐mitochondria O_2_ transport directly constrains the muscle bioenergetic capacity. In a complementary study, our group used phosphorus‐31 magnetic resonance spectroscopy (^31^P‐MRS) to quantify mitochondrial oxidative capacity by measuring the post‐exercise phosphocreatine (PCr) recovery time constant (τPCr). Young muscle exhibited rapid PCr recovery (τPCr ~37 s), whereas aged muscle showed a longer PCr recovery (τPCr ~52 s), indicating age‐associated impaired oxidative phosphorylation. This decline likely reflects multiple contributing factors, including reduced mitochondrial content, impaired oxygen delivery, and altered mitochondrial organization and function, rather than respiratory supercomplex instability alone. Similarly, using Arterial Spin Labeling (ASL) MRI, we found that in healthy adults, older age correlates with lower resting muscle perfusion as well as lower oxidative capacity (longer τPCr), suggesting that reduced perfusion and impaired oxidative metabolism likely interact through reciprocal mechanisms (Adelnia et al. 2019). Reduced oxygen availability may constrain mitochondrial performance, while mitochondrial dysfunction may simultaneously impair tissue energetics and vascular responsiveness. These findings suggest that impaired oxygen availability, reduced mitochondrial content, and late‐life muscle atrophy may be mechanistically linked. However, the temporal sequence of these changes remains uncertain. Longitudinal studies will be required to determine whether oxygen limitation precedes mitochondrial decline, follows it, or interacts with it bidirectionally during aging.

Proteomic analyses of skeletal muscle in healthy individuals across a wide age‐range further support this notion, revealing a distinct reduction of mitochondrial proteins, specifically those involved in the tricarboxylic acid (TCA) cycle and electron transport chain, in the skeletal muscle of less active older adults (Ubaida‐Mohien et al. 2019). The reduction in mitochondrial proteins suggests that the “bioenergetic ceiling” in aging is lowered not just by oxygen availability, but also by a reduction of mitochondrial content that impairs efficient oxygen utilization.

As discussed above, reduced mitochondrial content and impaired oxygen delivery are likely the dominant contributors to these impaired recovery kinetics. Age‐related “mild” uncoupling of mitochondria, whereby aging muscle increases proton leak (via Uncoupling Protein 3, UCP3) as a strategy to reduce ROS production, may further contribute, albeit at the cost of reduced ATP generation efficiency (Amara et al. 2007; Adelnia et al. 2019). Similarly, the stability of mitochondrial supercomplexes (also known as respirasomes) has been shown to decline with age, and this age‐related structural instability may further exacerbate electron leak and ROS production. The relative contribution of reduced mitochondrial content, impaired oxygen delivery, and mild uncoupling/supercomplex instability to the observed PCr recovery kinetics remains to be formally quantified (Genova and Lenaz 2015; Maranzana et al. 2013; Genova and Lenaz 2013).

## 6 Molecular Maladaptation Mechanisms Associated with HIF1 Sensor

When the physiological oxygen cascade fails and oxygen in cells drops, cells undergo a reprogramming survival strategy governed by the Hypoxia‐Inducible Factor (HIF) system. In normoxia, Prolyl Hydroxylase Domain (PHD) enzymes utilize oxygen, 2‐oxoglutarate, and iron (in its oxidation state Fe^2+^) to hydroxylate HIF‐1α, targeting it for proteasomal degradation via the von Hippel–Lindau (VHL) complex (Kaelin Jr. and Ratcliffe 2008; Schofield and Ratcliffe 2004). Kinetically, the K_m_ for oxygen is relatively high (~230–250 μM), implying that the rate of hydroxylation is directly limited by oxygen availability (Hirsila et al. 2003). HIF1 is comprised of two subunits: HIF‐1α, inducible by hypoxia, and HIF‐1β (or ARNT), expressed constitutively (Semenza 2012). As oxygen levels fall, HIF‐1α is stabilized and coordinates adaptive transcriptional programs.

Hypoxia sensing and angiogenic adaptation require a compliant and responsive vascular framework. When vascular structure and perfusion dynamics are impaired, compensatory signaling becomes inefficient or disordered. Dysregulated perfusion regulation represents a distinct and underappreciated failure mode of the aging oxygen cascade, linking extracellular matrix remodeling, vascular stiffness, and maladaptive oxygen sensing. Aging is characterized by a failure of these dynamic perfusion systems, namely the capacity to rapidly, locally, and reversibly match blood flow to metabolic demand (Herman et al. 2026). Even when vessels are structurally present, age‐related alterations in endothelial signaling, pericyte function, and ECM composition blunt vasomotor responsiveness, producing spatial and temporal heterogeneity in tissue perfusion and patchy areas of hypoxia. Progressive ECM remodeling through fibrosis, collagen crosslinking, and increased stiffness alters endothelial mechanotransduction, impairing shear‐stress sensing and uncoupling oxygen demand from vascular response. Thus, while oxygen delivery may be sufficient, it might not be functionally distributed and delivered at the local level, due to impaired vasomotor responsiveness, which prevents the swift redistribution of flow as needed to the most metabolically active regions. This loss of control generates fluctuating microregional hypoxia rather than uniform oxygen deprivation. In the context of Oxygenaging, this dynamic instability amplifies maladaptive HIF‐1 cycling, impairs angiogenic efficiency, and increases cellular vulnerability to oxidative injury during periods of reoxygenation.

Microvascular rarefaction is commonly interpreted as caused by defective angiogenesis, not fully highlighting the fact that endothelial cells themselves age. Endothelial senescence, mitochondrial dysfunction, and altered HIF signaling may limit the capacity to mount an appropriate response to hypoxia. Therefore, capillary rarefaction represents an active failure of endothelial adaptation, not merely a deficit of pro‐angiogenic stimuli. Senescent endothelial cells are often characterized by impaired Vascular Endothelial Growth Factor Receptor 2 (VEGFR2) signaling and downstream angiogenic responses (Berenjabad et al. 2022; Regina et al. 2016) that normally induce sprouting, migration, and proliferation (Schaaf et al. 2019). When Vascular Endothelial Growth Factor (VEGF)‐VEGFR2 signaling is blunted, endothelial cells lose the phenotypic flexibility required for angiogenesis and display reduced tip–stalk plasticity with altered cytoskeletal dynamics that limit sprouting even in the presence of stabilized HIF‐1α (Pasut et al. 2021). In parallel, senescent endothelial cells are also associated with mitochondrial dysfunction, telomere damage, and senescence‐associated secretory phenotype (SASP), which further suppresses angiogenic capacity (Bloom et al. 2023). Collectively, in Oxygenaging microvascular loss reflects not only an insufficient delivery of oxygen but a failure of the aging endothelium to translate hypoxic signaling into functional vascular remodeling.

**FIGURE 2:**
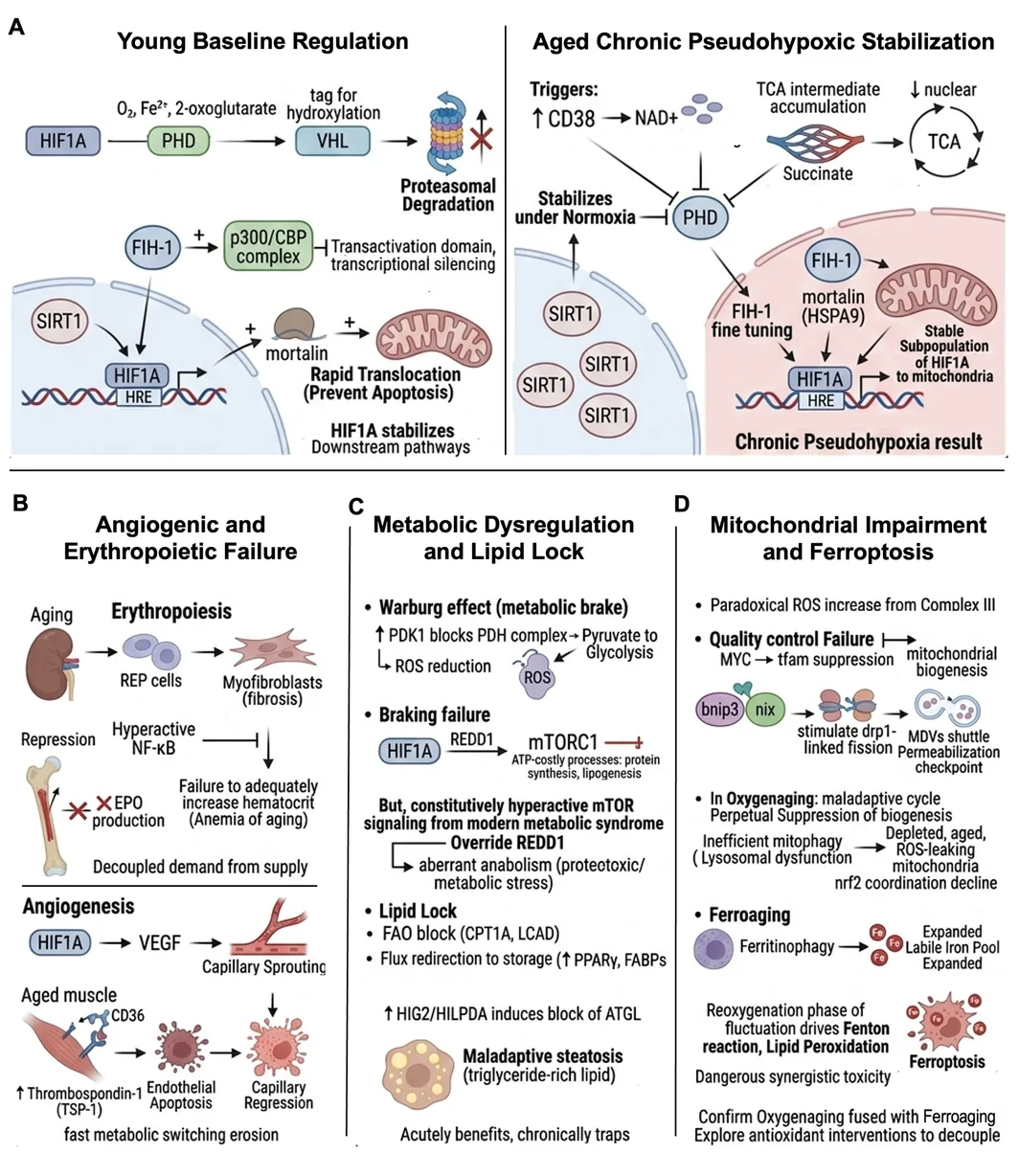
Molecular maladaptation in Oxygenaging. The cellular Hypoxia‐Inducible Factor (HIF) survival system becomes severely maladaptive with age. (A) Chronic Pseudohypoxia. In youth, Prolyl Hydroxylase Domains (PHDs) degrade HIF‐1α, and VHL‐dependent proteasomal degradation keeps HIF‐1α levels low. In Oxygenaging, two mechanistically distinct routes converge to stabilize HIF‐1α under normoxia: Declining NAD^+^ reduces SIRT1 activity and VHL expression (independent of PHDs), while separately, succinate accumulation directly inhibits PHD catalytic activity, driving a state of Chronic Pseudohypoxia. (B) Angiogenic and Erythropoietic Collapse. Renal EPO production fails due to pathological cellular transdifferentiation and NF‐κB repression (the “anemia of aging”). Concurrently, elevated Thrombospondin‐1 (TSP‐1) induces endothelial apoptosis, overriding VEGF signaling and locking tissues in vascular rarefaction. (C) Metabolic Dysregulation. HIF‐1α normally acts as a metabolic brake by shunting to glycolysis and inhibiting mTORC1. In aging, constitutively hyperactive mTORC1 overrides this brake, driving aberrant anabolism and proteotoxic stress. Simultaneously, HIF‐1α inhibits lipolysis (creating a “Lipid Lock”), driving maladaptive steatosis. (D) Mitochondrial Failure and Ferroptosis. HIF‐1α suppresses mitochondrial biogenesis, which, combined with impaired mitophagy, leaves a pool of depleted, ROS‐leaking mitochondria. This hypoxic dysfunction intersects with iron dysregulation (Ferroaging): An expanded labile iron pool (LIP) catalyzes the Fenton reaction during reoxygenation, which may trigger fatal ferroptosis.

The response of HIFs is not limited to hypoxia/anoxia but is sensitive to oxygen fluctuation, acting proactively as a “metabolic homeostat” (Arias et al. 2025; Semenza 2011). However, regulation extends beyond PHDs. Factor Inhibiting HIF‐1 (FIH‐1) provides a secondary layer of fine‐tuning by uncoupling HIF‐1α stabilization from transcriptional activation. Even when HIF‐1α accumulates, FIH‐1 hydroxylates its transactivation domain, blocking recruitment of the co‐activators p300/CBP that are required for gene transcription, thereby functionally silencing the pathway (Schodel et al. 2010; Volkova et al. 2022). Furthermore, HIF‐1α and sirtuin 1 (SIRT1) exhibit a mutual regulation loop: SIRT1 deacetylates and activates HIF‐1α, making SIRT1‐dependent fine‐tuning indispensable for coordinating metabolic adjustments (Lim et al. 2010; Ryu et al. 2019). Additionally, a subpopulation of HIF‐1α can undergo rapid, chaperone‐mediated translocation to the mitochondria (facilitated by the chaperone mortalin, HSPA9) to stabilize membrane potential and prevent apoptosis (Mylonis et al. 2017; Li, Zhou, et al. 2019). Rather than static defense, this functional HIF response represents a cascading integration of systemic supply, metabolic tuning, and mitochondrial shielding, all of which become maladaptive in Oxygenaging. This framework is visually summarized in Figure 2A.

### 6.1 Angiogenic and Erythropoietic Failure

The primary adaptive response to hypoxia is to restore oxygen delivery (DO_2_) through HIF‐dependent transcription of genes encoding erythropoietin (EPO) and VEGF (Semenza 2012). This coordinated erythropoietic and angiogenic program is essential for tissue maintenance, but it becomes markedly impaired with age (Ungvari, Tarantini, Donato, et al. 2018; Carmeliet 2005). While HIF‐1α predominantly mediates acute metabolic adaptation to hypoxia, its isoform HIF‐2α drives the more sustained erythropoietic response. Both isoforms are regulated by PHD‐ and VHL‐dependent hydroxylation, yet they differ in tissue distribution and kinetic profile, with HIF‐2α supporting longer‐term adaptation. The impairment of this supply chain was proposed to contribute to the “anemia of aging” and a failure to adequately increase the hematocrit despite declining arterial oxygen partial pressure (P_a_O_2_) (Ershler et al. 2005; Ferrucci et al. 2007; Recalcati et al. 2015; Stauder et al. 2018). Aging kidneys produce less EPO in response to hypoxia; this defect is linked to the fact that renal erythropoietin‐producing (REP) cells, specialized peritubular fibroblasts responsible for EPO synthesis, undergo a pathological transdifferentiation into myofibroblasts during aging and fibrosis (Souma et al. 2013; Koury and Haase 2015). Mechanistically, this transformation renders them non‐functional, resulting in impaired HIF‐2α signaling due to hyperactive signaling of NF‐κB (which acts as a transcriptional repressor of EPO) and a subsequent failure to produce EPO for a given degree of hypoxia (Souma et al. 2016). While hematopoietic stem cells become less sensitive to EPO stimulation, further compounding the deficit, it is this failure of the REP cells that primarily decouples molecular demand from systemic supply. Simultaneously, the angiogenic potential of the microvasculature collapses. While HIF‐1α normally drives VEGF‐mediated capillary sprouting, at least in skeletal muscle and likely also in other tissues, the levels of the angiostatic factor Thrombospondin‐1 (TSP‐1) increase with aging (Audet et al. 2013). TSP‐1 signals via the CD36 receptor to actively promote endothelial apoptosis and capillary regression, overriding the VEGF signal. This anti‐angiogenic “failure” effectively locks the tissue in a state of vascular rarefaction. Cellular survival during acute oxygen fluctuations is guaranteed by immediate non‐genomic protection, such as rapid metabolic switching and ROS signaling, yet this fast adaptability has been hypothesized to erode with aging (Figure 2B) (Pomatto and Davies 2017).

### 6.2 Metabolic Dysregulation and Lipid Lock

To ensure survival when supply cannot be restored, HIF‐1α actively suppresses high‐energy anabolic processes to match metabolic demand to the failing supply. This “metabolic brake” relies on the increased levels of Pyruvate Dehydrogenase Kinase 1 (PDK1), which phosphorylates and thereby inactivates the Pyruvate Dehydrogenase complex (PDH). Such inhibition prevents pyruvate from entering the TCA cycle, shunting glucose‐derived metabolites toward glycolysis (the Warburg effect). By reducing mitochondrial oxygen consumption, this PDK1‐mediated shunt serves as a central enzymatic step for energy conservation and the prevention of toxic ROS production under hypoxia; if the PDK1 brake fails, continued oxidative phosphorylation under severe hypoxia causes massive electron leak and ROS accumulation at Complex III (Fukuda et al. 2007; Kim et al. 2006; Papandreou et al. 2006). Crucially, HIF‐1α also inhibits the mechanistic Target of Rapamycin Complex 1 (mTORC1) via the induction of REDD1 (Regulated in development and DNA damage responses 1) (Wouters and Koritzinsky 2008; Brugarolas et al. 2004). mTORC1 inhibition arrests protein synthesis, the most ATP‐costly cellular process, and suppresses lipogenesis to conserve resources (Mylonis et al. 2019).

In Oxygenaging, this braking mechanism fails due to constitutively hyperactive mechanistic Target of Rapamycin (mTOR) signaling, a central regulator of aging and metabolism, and its deregulation is a key driver of age‐related pathology (Kennedy and Lamming 2016; Laplante and Sabatini 2012). This is often driven by nutrient excess (especially prevalent in the context of modern metabolic syndrome and sedentary lifestyles) or loss of sensitivity to nutrient withdrawal (e.g., insulin resistance) (Salminen et al. 2016), resulting in a HIF‐1α attempting to stop conserving energy, while hyperactive mTOR signals “go” for growth. This molecular conflict leads to aberrant anabolism, where cells continue to consume energy and produce proteins despite insufficient oxygen, ultimately resulting in severe proteotoxic and metabolic stress (Salminen et al. 2016).

This metabolic reprogramming extends to lipid homeostasis. To minimize oxygen consumption, HIF‐1α actively suppresses mitochondrial fatty acid β‐oxidation (FAO) by reducing the levels of medium‐ and long‐chain acyl‐CoA dehydrogenases (MCAD and LCAD) and inhibiting Carnitine Palmitoyltransferase 1A (CPT1A), the rate‐limiting enzyme for fatty acid entry into the mitochondria, as reported in cancer models (Huang et al. 2014). In parallel, HIF‐1α redirects metabolic flux toward lipid storage by increasing the production of PPARγ and fatty acid‐binding proteins (FABPs) to facilitate uptake (Bensaad et al. 2014). To prevent the mobilization of these stored lipids, HIF‐1α induces the expression of Hypoxia‐Inducible Lipid Droplet Associated Protein (HILPDA/HIG2). HILPDA binds to and inhibits Adipose Triglyceride Lipase (ATGL). This effectively creates a lipid lock that blocks lipolysis and sequesters fatty acids, driving the accumulation of triglyceride‐rich lipid droplets within hepatocytes, which is clinically manifested as liver steatosis (fatty liver) (Padmanabha Das et al. 2018). In youth, this lipid lock is a beneficial acute mechanism that protects the cell from acute oxidative stress and lipotoxicity. Instead, in the chronic context of Oxygenaging, persistent activation of these pathways may contribute to maladaptive lipid accumulation and steatosis. Hepatic de novo lipogenesis (DNL) and age‐related carnitine insufficiency, which can reduce β‐oxidation, are both implicated in the development of liver steatosis during aging (Noland et al. 2009). Steatosis is multifactorial and involves insulin resistance, increased de novo lipogenesis, altered nutrient sensing, and mitochondrial dysfunction. Thus, the HIF‐mediated “lipid lock” described here should be viewed as one potential contributor to a broader metabolic network promoting lipid accumulation in aging (Figure 2C).

### 6.3 Mitochondrial Integrity Impairment and Ferroptosis

The last adaptive mechanism we discuss is an oxidative shield mediated by mitochondrial quality control. Mitochondria have evolved to function not just as powerhouses, but as signaling organelles that communicate cellular stress (Chandel 2014). It has also been found that mitochondria are reshaped by diminished oxygen, enabling mitochondria to transmit information about oxygen levels to key transcriptional regulators (Fuhrmann and Brune 2017). For example, hypoxia increases mitochondrial ROS release from Complex III, which acts as a rapid signal for adaptation (Chandel et al. 1998; Guzy et al. 2005).

HIF‐1α mechanisms aim to shield the cell from potential redox collapse by regulating the mitochondrial pool. HIF‐1α antagonizes MYC activity; since MYC normally stimulates mitochondrial transcription factor A (TFAM) to drive mitochondrial biogenesis, HIF‐1α‐mediated inhibition of MYC (often via direct binding or competition for co‐factors like SP1) effectively suppresses new mitochondrial formation, thereby limiting oxygen consumption and ROS generation (Zhang et al. 2007). Because TFAM is essential for mitochondrial DNA (mtDNA) maintenance, transcription, and replication, its decline compromises mitochondrial genome stability and limits oxidative phosphorylation (Li et al. 2005). Concurrently, HIF‐1α activates the receptor proteins BNIP3 and NIX (also known as BNIP3L, a distinct but related mitophagy receptor) to trigger the selective autophagy of mitochondria (mitophagy) and stimulates dynamin‐related protein 1 (DRP1)‐linked fission, which fragments the mitochondrial network to facilitate the clearing of dysfunctional organelles that leak ROS (Bellot et al. 2009; Wan et al. 2014; Zhang and Ney 2009). Emerging evidence also points to the release of Mitochondrial‐Derived Vesicles (MDVs) as a first line of defense before whole‐organelle mitophagy. MDVs shuttle damaged cargo to lysosomes for degradation, a process that is critical for quality control but declines with age (Sugiura et al. 2014; Picca et al. 2023). The permeabilization of the mitochondrial membrane is a critical checkpoint in cell death, regulated by this precise machinery (Kroemer et al. 2007).

In Oxygenaging, the chronic decline of nuclear NAD^+^ driven by the age‐related increased abundance of the NADase CD38 (Camacho‐Pereira et al. 2016), leads to a state of “pseudohypoxia,” where HIF‐1α is stabilized not by low oxygen, but by metabolic dysfunction (Gomes et al. 2013). This pseudohypoxic state can arise through at least two mechanistically distinct, convergent routes. First, declining NAD^+^ levels reduce SIRT1 activity, lowering VHL expression and thereby impairing VHL‐mediated proteasomal degradation of HIF‐1α, independently of PHD hydroxylation status. Second, age‐related declines in TCA cycle enzyme activity (such as that of succinate dehydrogenase, SDH) drive accumulation of succinate, which directly inhibits PHD catalytic activity. Although mechanistically distinct, both routes converge on the same outcome, HIF‐1α stabilization under normoxia (“Chronic Pseudohypoxia”) (Gomes et al. 2013; Selak et al. 2005). This chronic activation creates a maladaptive cycle. Mitochondrial biogenesis is suppressed by the MYC/TFAM axis, while mitophagy becomes inefficient due to lysosomal dysfunction (Lopez‐Otin et al. 2023), leading to a pool of depleted, aged, ROS‐leaking mitochondria and mitochondrial fragments. Furthermore, the coordination with NRF2 (the master antioxidant regulator) declines with age, leaving the cell vulnerable to unbuffered oxidative damage (Schmidlin et al. 2019).

The intersection of dysregulated oxygen sensing and iron homeostasis poses a unique risk in aging, the iron‐dependent form of cell death distinct from apoptosis named ferroptosis. Aging tissues often accumulate non‐transferrin‐bound iron despite systemic anemia. Since PHDs require iron as a co‐factor, iron dysregulation directly impacts oxygen sensing, while 2‐oxoglutarate/α‐KG levels decline with age, leading to a drop in α‐KG to succinate ratio that further inhibits PHDs. Moreover, during the reoxygenation phases of hypoxic fluctuation, the presence of labile iron can catalyze the Fenton reaction, drive lipid peroxidation, and trigger ferroptosis (Stockwell et al. 2017; Dixon et al. 2012). Recent studies have identified NCOA4 (nuclear receptor coactivator 4) as a key regulator of this process via “ferritinophagy” (the autophagic degradation of ferritin). In aging, dysregulated NCOA4 activity can release excess iron into the Labile Iron Pool (LIP), increasing susceptibility to ferroptosis under hypoxic stress (Jenkins et al. 2020; Santana‐Codina et al. 2022). In turn, ferroptosis creates a dangerous synergistic toxicity in the context of “Oxygenaging”: the hypoxic tissue requires HIF‐1α stabilization, but iron dysregulation disrupts this adaptive response. Aberrant iron accumulation can inappropriately maintain PHD activity and degrade HIF‐1α, while it simultaneously primes the cell for ferroptosis during re‐oxygenation phases. Thus, the aged cell is caught between hypoxic energy failure and hyperoxic iron‐mediated death. This mechanism confirms that “Oxygenaging” is inextricably fused with “Ferroaging,” a critical intersection that we identify as a priority for future exploration, specifically to determine how antioxidant interventions can decouple these pathways during therapy (Figure 2D).

## 7 Transcriptomic and Epigenomic Responses to Oxygenaging

The Oxygenaging framework does not imply that exceptional longevity should be determined primarily by genes directly involved in oxygen transport. Rather, oxygen homeostasis emerges from the coordinated function of multiple physiological systems. Accordingly, genetic pathways associated with exceptional longevity may preserve oxygen homeostasis indirectly through effects on stress resistance, mitochondrial function, metabolic efficiency, vascular health, and adaptive capacity. This perspective is consistent with the observation that many longevity‐associated loci influence resilience mechanisms rather than specific components of the oxygen transport machinery (Deelen et al. 2019).

### 7.1 Dysregulation of the RNA Splicing Machinery

The metabolic reprogramming in response to hypoxia also relies on alternative splicing, which acts as a global transcriptomic modifier. Hypoxia does not only alter the quantity of mRNA, but it creates a specific “hypoxic RNA splicing landscape” with specific splicing isoform expression, as the example of the Pyruvate Kinase M (PKM) splicing switch. Under hypoxic metabolic stress, HIF‐1α cooperates with MYC to increase the expression of Heterogeneous Nuclear Ribonucleoproteins (hnRNPs), particularly hnRNP A1 and hnRNP A2, which promote inclusion of exon 10 over exon 9 in *PKM* mRNA. This shift favors expression of the PKM2 isoform over the constitutively active PKM1 (David et al. 2010). This splice‐switching event is metabolically decisive: whereas PKM1 drives oxidative phosphorylation, PKM2 slows the flux of glycolytic intermediates, enforcing a “metabolic brake” that supports redox balance and antioxidant defense (Gallego‐Paez et al. 2017).

It was found that hypoxia regulates the splicing factor SRSF6 through the inclusion of a poison cassette exon (PCE), a noncoding exon containing a premature termination codon that targets the transcript for nonsense‐mediated decay (NMD) (de Oliveira Freitas Machado et al. 2023). Transient downregulation of SRSF6 during hypoxia appears adaptive, rapidly reprogramming global splicing to suppress translation of non‐essential proteins and conserve energy. However, in aging this regulatory mechanism may become dysregulated, leading to maladaptive alterations in RNA processing.

Global intron retention (IR) serves as a rapid, reversible mechanism to suppress the translation of non‐essential transcripts during cellular stress and differentiation (Wong et al. 2013). Elevated intron retention is now recognized as a distinct molecular signature of aging across multiple tissues (Adusumalli et al. 2019), potentially contributing to the accumulation of aberrant proteins and neoepitopes that trigger surveillance and clearance from immune cells. Hypoxia engages similar IR‐based strategies, including intron retention in EIF2B5 that inhibits translation (Brady et al. 2017), and induces widespread alternative splicing changes across the transcriptome (Han et al. 2017), collectively shaping a distinct “hypoxic splicing landscape”. Single‐cell transcriptomic analyses suggest that aging introduces significant heterogeneity in this response and that transcriptomic “noise” in aged cells can disrupt the elevation of hypoxic‐specific isoforms, compromising the efficacy of this metabolic brake (Enge et al. 2017).

### 7.2 Senescence‐Like Accumulation of Aberrant Methylation

Oxygen availability also directly dictates the “ticking” of the epigenetic clock. Recently, we showed that in C57BL/6 female mice, an intervention of 2:30 min/cycle IH for 8 h/day for 4 weeks induced up to a 5‐month epigenetic age acceleration in lung, spleen, and heart in old mice only (not in adult mice), and that this age acceleration reversed upon return to normoxia after 4 weeks (Donega et al. 2026). DNA methylation, a core component of the epigenetic landscape and the basis for “aging clocks” (Horvath 2013; Lu et al. 2019), is actively regulated by Ten‐Eleven Translocation (TET) enzymes. TET enzymes (TET1‐3) are dioxygenases that facilitate DNA demethylation by converting 5‐methylcytosine (5mC) to 5‐hydroxymethylcytosine (5hmC). Oxygen is an essential co‐substrate for this reaction. Hypoxia acts as a direct inhibitor of TET activity, independent of enzyme abundance: under hypoxic conditions, this substrate limitation leads to a “hypermethylation block” at specific gene promoters, often silencing tumor suppressor genes and mimicking the age‐associated epigenetic drift (Thienpont et al. 2016). Oxygen also regulates histone demethylases, specifically Lysine‐specific Demethylases (KDMs) such as KDM5A and KDM6A, which are also JmjC domain‐containing dioxygenases dependent on oxygen and 2‐oxoglutarate (Chakraborty et al. 2019; Batie et al. 2019). The hypoxic inhibition of KDMs leads to hypermethylated histone marks (e.g., H3K4me3, H3K27me3), condensing chromatin and locking cells in a senescent‐like transcriptional state (Chakraborty et al. 2019; Batie et al. 2019). In this context, chronic intermittent hypoxia, such as that seen in obstructive sleep apnea, has been shown to accelerate epigenetic aging markers in blood DNA, a process potentially reversible with adherent CPAP therapy (Cortese et al. 2022).

The Oxygenaging decline in tissue pO_2_ may physically limit the catalytic efficiency of TET and KDM enzymes, contributing to the progressive accumulation of aberrant methylation patterns characteristic of cellular senescence (Horvath and Raj 2018). The core clock proteins (BMAL1/CLOCK) directly regulate HIF‐1α expression, creating a bidirectional feedback loop that modulates the circadian clock (Peek et al. 2017). In older organisms, this sensing mechanism is disrupted by circadian desynchronization, metabolic interference from accumulated succinate (Mills et al. 2016), and toxic metal displacement of iron from the PHD active site (Aschner et al. 2023).

## 8 Gerotherapeutic Oxygen‐Modulation Interventions

An important implication of the Oxygenaging framework is that many interventions known to influence biological aging may act, at least in part, by modifying resilience across the oxygen cascade. Exercise enhances oxygen transport and utilization at virtually every level of the cascade, whereas smoking, obesity, diabetes, and chronic physical inactivity impair multiple components simultaneously (Hawley et al. 2014; Van Gaal et al. 2006; Dixon and Peters 2018). Similarly, interventions such as caloric restriction, rapamycin, and metformin influence the balance between cellular oxygen utilization, energetic demand, and mitochondrial adaptation (Lopez‐Lluch and Navas 2016; Johnson et al. 2013; Barzilai et al. 2016). Thus, Oxygenaging may provide a physiological framework for understanding how diverse geroprotective interventions converge on common pathways that preserve functional capacity.

An implication is the distinction between chronological and biological aging. Individuals of similar chronological age exhibit marked heterogeneity in physiological reserve and functional capacity. From an Oxygenaging perspective, such heterogeneity may reflect differences in the ability to preserve oxygen homeostasis across the cascade. Exceptional agers may therefore represent individuals who maintain resilience at multiple levels of oxygen transport, diffusion, utilization, and sensing, thereby delaying the emergence of functional decline despite advanced age.

Emerging Oxygenaging therapies modulate this “Supply–Demand‐Quality Control” axis to extend healthspan. Table 2 summarizes how these interventions target distinct nodes of this homeostatic network, highlighting the most recent findings.

**TABLE 2:** Emerging oxygenaging interventions.

Intervention	Protocol	Results	Mechanism	References
Intermittent hypoxia (IH)	Low‐dose (mild 9%–16% O_2_) versus severe/chronic high‐dose protocols	Low‐dose regimens promote neuroplasticity and functional benefits, whereas chronic exposure induces systemic oxidative injury	Transcriptionally upregulates neurotrophic factors (e.g., BDNF) and associated receptors (TrkB) while avoiding maladaptive inflammatory stress	(Navarrete‐Opazo and Mitchell 2014)
	10–15 cycles of acute IH (9%–16% O_2_) paired with targeted motor tasks	Augmentation of respiratory long‐term facilitation (LTF) and enhanced limb motor function post‐spinal cord injury	Activation of 5‐HT signaling and downstream BDNF synthesis, facilitating robust synaptic strengthening	(Gonzalez‐Rothi et al. 2015; Mitchell et al. 2001)
Hyperbaric oxygen therapy (HBOT)	100% O_2_ at > 2.0 ATA (60 sessions, 90 min each)	Induction of significant telomere elongation (> 20%) and targeted reduction of the senescent T‐cell population (~37%)	Rapid ambient pressure reduction mimics hypoxic signaling, activating HIF‐1α‐mediated regenerative pathways and telomerase activity (Hyperoxic‐Hypoxic Paradox)	(Hachmo et al. 2020)
	100% O_2_ at > 2.0 ATA	Significant clearance of senescent cutaneous cells and a systemic reduction in circulating SASP factors.	Reversal of hypoxia‐induced immunosuppression; hyperoxic fluctuations restore OXPHOS in Natural Killer (NK) cells to eliminate senescent targets	(Hachmo et al. 2021)
	100% O_2_ at 2.0 ATA (60 sessions)	Significant gains in attention and processing speed, coupled with enhanced regional cerebral perfusion	Induction of VEGF‐mediated “normalized” angiogenesis and mobilization of endothelial progenitor cells to restore microvascular networks	(Hadanny et al. 2020)
Intermittent hypoxic‐hyperoxic treatment (IHHT)	Brief hypoxic (10%–14% O_2_) and hyperoxic (30%–40% O_2_) intervals (30–45 min)	Enhanced global cognitive scores (e.g., DemTect), surpassing outcomes achieved by multimodal physical training alone	Hyperoxia provides the ROS signaling Trigger, while hypoxia drives adaptive transcriptional expression and mitochondrial selection (Driver)	(Bayer et al. 2017)
	Analysis of alternating hypoxic‐hyperoxic conditioning	Restoration of mitochondrial bioenergetics and enhanced systemic stress resilience	Controlled ROS signaling coupled with HIF‐1α‐mediated metabolic reprogramming to clear damaged organelles via mitophagy	(Salvagno et al. 2022)

### 8.1 Intermittent Hypoxia for Restoring Neural and Vascular Plasticity

Intermittent Hypoxia (IH) mainly targets the sensing machinery of this oxygen homeostatic network. Unlike chronic hypoxia, carefully dosed IH is designed to target neural and vascular plasticity, acting as a hormetic stressor (Navarrete‐Opazo and Mitchell 2014). Translational work shows that acute IH augments motor recovery from spinal injury when paired with task‐specific practice: low‐dose IH stabilizes HIF‐1α and activates NRF2 to transcriptionally increase the expression of genes encoding neurotrophic factors such as BDNF and its receptor TrkB. This enhances synaptic strengthening and induces long‐term facilitation (LTF) of motor output without causing tissue injury (Gonzalez‐Rothi et al. 2015). Such protocols can induce a sustained increase in respiratory motor output driven by serotonin‐dependent synaptic strengthening (Mitchell et al. 2001). In motor‐incomplete spinal cord injury, a randomized trial uncovered that daily IH improved over‐ground walking speed and endurance compared with sham exposure (Navarrete‐Opazo et al. 2017).

Hematologic responses to IH are highly variable; while some regimens alter red‐cell indices, others show no durable shift, consistent with the view that the primary benefit of low‐dose IH lies in neuromodulation and systemic vascular plasticity (achieved via shear stress fluctuations and episodic VEGF/eNOS pulses that trigger beneficial vessel remodeling without the chronic leakage associated with continuous hypoxia) rather than erythrocytosis (Viscor et al. 2018). Systemic adaptations to hypoxia are highly dependent on context, with beneficial or maladaptive responses emerging as a function of the hypoxic dose, defined by its severity, duration, and intermittency (Verges et al. 2015). Most evidence defining this therapeutic window comes from animal studies or human disease populations rather than healthy older adults. Thus, while low‐dose IH is mechanistically promising, its efficacy and safety as a gerotherapeutic intervention in aging populations remain to be established.

It is important to distinguish therapeutic IH from the pathological intermittent hypoxia observed in Obstructive Sleep Apnea (OSA). While both involve fluctuations in oxygen, OSA is characterized by high‐frequency, uncontrolled cycling (dozens of events per hour) often accompanied by hypercapnia and sleep fragmentation, which drives chronic sympathetic hyperactivity and cardiovascular damage. Indeed, the vascular impact of hypercapnia is often overlooked, yet recent evidence demonstrates that intermittent hypercapnia alone (independent of hypoxia) can drive persistent increases in mean arterial pressure and long‐term changes in cardiovascular control (Shing et al. 2025). The key distinction lies in the signaling pathway: pathologic high‐frequency IH preferentially activates inflammatory NF‐κB signaling, whereas therapeutic low‐dose IH activates adaptive NRF2 and HIF‐1α pathways without overwhelming antioxidant defenses (Navarrete‐Opazo and Mitchell 2014; Serebrovskaya and Xi 2016). In addition, therapeutic IH utilizes a low‐dose approach (typically few, longer cycles with controlled reoxygenation) that targets the therapeutic window to elicit adaptive plasticity (hormesis) without crossing the threshold into oxidative injury (Mateika et al. 2015).

### 8.2 Hyperbaric Oxygen Therapy for Clearing Senescent Cells

Hyperbaric Oxygen Therapy (HBOT) primarily targets the oxygen supply deficit but utilizes a unique sensing mechanism. By supplying 100% O_2_ at > 1.5 atm absolute pressure (ATA), it dissolves sufficient oxygen to bypass hemoglobin limits. Crucially, HBOT relies on the “Hyperoxic‐Hypoxic Paradox”: the rapid return to normoxia from hyperoxia is sensed by the cell as a relative hypoxic event. It is critical to distinguish this from the “Normobaric Oxygen Paradox” (see IHHT below). While both rely on relative fluctuation, HBOT utilizes a pressure drop (from > 1.5 ATA to 1 ATA) to maximize the amplitude of the signal.

The “Hyperoxic‐Hypoxic Paradox” leverages the drastic absolute pressure drop from > 1.5 ATA to sea level to maximize the HIF‐1α signal amplitude without incurring actual tissue hypoxia. This fluctuation triggers HIF‐1α stabilization and transcriptionally increases production of VEGF without the metabolic consequences of true hypoxia (Fu et al. 2022). Furthermore, one small prospective study reported increases in telomere length in immune‐cell subsets following HBOT (Hachmo et al. 2020). However, these findings should be interpreted cautiously given the limited sample size, lack of randomized sham control, and uncertainty regarding the extent to which peripheral blood telomere changes reflect broader biological rejuvenation. In particular, evidence that HBOT modifies validated biomarkers of biological aging in healthy older adults remains preliminary and requires confirmation in larger randomized controlled trials.

Clinically, these cellular mechanisms translate into functional improvements. Randomized controlled trials have demonstrated that HBOT induces significant cognitive enhancement in healthy older adults, specifically by improving cerebral blood flow and restoring brain microstructure (Hadanny et al. 2020). Furthermore, HBOT has been shown to induce significant telomere elongation (> 20%) in immune cells, acting as a measurable marker of cellular rejuvenation (Hachmo et al. 2020). Randomized trials combining HBOT (2286 participants: 1103 allocated to HBOT and 1153 to control) with radiotherapy have shown improved tumor control but also increased late radiation‐related toxicity (Bennett et al. 2018). Importantly, independent safety analyses demonstrate that HBOT itself does not promote tumor growth or recurrence, indicating no evidence that the therapy is oncogenic (Feldmeier et al. 2003). While both hypoxia and HBOT can stimulate angiogenesis, they trigger distinct morphological outcomes: hypoxia drives the formation of chaotic, leaky, and immature vessels via uncontrolled HIF‐1α overexpression (Carmeliet and Jain 2011), whereas HBOT drives normalized, structured angiogenesis. This distinction arises because HBOT provides the oxidative substrate required for collagen maturation and basement membrane stabilization (two processes that are arrested in pure hypoxia) while recruiting bone‐marrow‐derived endothelial progenitor cells (EPCs) to form patent, functional capillaries (Moen and Stuhr 2012; Thom et al. 2006). Additionally, the restoration of immune surveillance enables clearance of senescent cells, which typically accumulate due to age‐related immunosenescence (Hachmo et al. 2021). Ultimately, the clearance of senescent cells is a critical strategy for ameliorating age‐related phenotypes and pathologies (Childs et al. 2015; Kirkland and Tchkonia 2017).

### 8.3 Intermittent Hypoxic‐Hyperoxic Training for Optimizing Mitochondrial Bioenergetics

Intermittent Hypoxic‐Hyperoxic Training (IHHT) targets mitochondrial quality control. This protocol alternates hypoxic bouts with brief hyperoxia. A crossover study in healthy volunteers detected that hyperoxia followed by normoxia increased erythropoietin levels during the delayed recovery window (beginning ~8 h after the last hyperoxic exposure and peaking between 24 and 36 h) when oxygen tension returns abruptly from supraphysiological levels back to baseline, creating a relative drop in tissue PO_2_ that the kidney interprets as a hypoxic signal. This delayed rebound illustrates the “Normobaric Oxygen Paradox” (Balestra et al. 2006), where the return to normoxia from hyperoxia is sensed by the cell as a relative hypoxic event, triggering early HIF‐1α accumulation within 4–6 h at the cellular level (Cimino et al. 2012).

Unlike HBOT, which relies on pressure to drive the paradox, IHHT relies on the rapidity of the FiO_2_ oscillation (hypoxia‐hyperoxia‐hypoxia). The hyperoxic phase acts as a ROS trigger, while the hypoxic phase acts as the adaptive driver (Salvagno et al. 2022). This oscillation forces mitochondrial selection via mitophagy, promoting the clearance of damaged organelles and selectively preserving those mitochondria capable of withstanding the ROS fluctuation (mitochondrial selection pressure), enhancing overall bioenergetics and ATP production (Serebrovskaya and Xi 2016; Bayer et al. 2017). However, clinical results remain heterogeneous, highlighting the need for precise dosing (Bayer et al. 2019). While some meta‐analyses report maximal oxygen uptake (VO_2_max) gains with IH training (Huang et al. 2023), others, restricted to parallel‐design trials, found no advantage over normoxic training, highlighting persistent heterogeneity in protocol duration and minimum FiO_2_ (Dorelli et al. 2025).

## 9 Translational Challenges and Therapeutic Windows

While the therapeutic potential of oxygen modulation is considerable, clinical translation warrants caution. The Oxygenaging framework reveals that aging organisms often lack homeostatic resilience to buffer extreme fluctuations in oxygen. The concept of hormesis relies on a dose–response curve that is likely shifted in aging; a stimulus that is adaptive in a young organism may be toxic in an older one due to the shrinking of the therapeutic window (Bondy 2023). This window is also likely modified by frailty, multimorbidity, and baseline cardiopulmonary reserve, so protocols derived from younger or disease‐specific cohorts cannot be assumed to translate directly to healthy older adults. This narrowing is, at least in part, driven by impaired NRF2 signaling, whereby reduced activation of antioxidant response elements (AREs, regulatory DNA sequences controlling cytoprotective gene expression) limits the capacity to mount an effective antioxidant response. As previously published, the efficiency of NRF2 nuclear translocation declines with aging, making aged tissues incapable of mounting the necessary antioxidant buffer against the ROS “trigger” of these therapies (Schmidlin et al. 2019).

In frail elderly populations, aggressive hypoxic or hyperoxic protocols could exacerbate oxidative stress, drive inflammation via NF‐κB activation (Eltzschig and Carmeliet 2011), or precipitate ferroptosis if the labile iron pool is already expanded: a convergence of pathologies that we define (but not explore here) as Ferroaging (Jenkins et al. 2020). Moreover, individual variability in the reflex increase in ventilation triggered by low arterial oxygen, mediated by peripheral chemoreceptors (the hypoxic ventilatory response, HVR), as well as in antioxidant capacity (e.g., glutathione levels) is high, meaning one‐size‐fits‐all protocols are likely to fail or cause harm (Serebrovskaya and Xi 2016).

Sex is another likely source of variability, as men and women differ in hemoglobin concentration, cardiopulmonary reserve, vascular function, iron homeostasis, and hypoxia‐responsive signaling. These differences may influence both the trajectory of Oxygenaging and the therapeutic window for oxygen‐modulating interventions.

Future research must therefore focus on: (I) Defining precise “therapeutic windows” tailored to biological age rather than chronological age; (II) Developing personalized protocols that monitor real‐time biomarkers of oxidative stress (e.g., F2‐isoprostanes) (Morrow 2005) and mitochondrial function (e.g., PBMC High‐Resolution Respirometry) (Chacko et al. 2014) during therapy; (III) Conducting large‐scale, randomized controlled trials that specifically stratify participants by frailty status to ensure safety. Only through such rigorous stratification can we transition these interventions from experimental promise to safe clinical practice.

## 10 Conclusions

In this review, we propose Oxygenaging as a physiological framework linking systemic oxygen transport, tissue oxygen availability, mitochondrial function, and molecular hallmarks of aging. Rather than representing a single causal mechanism, Oxygenaging highlights how progressive loss of resilience across the oxygen cascade may interact with and amplify established aging processes, with epigenetic drift, aberrant splicing, mitochondrial electron leaks, and macro‐scale oxygen transport deficits triggering a landslide of molecular maladaptations, ultimately contributing to functional decline and loss of physiological reserve. We suggest oxygen‐sensing pathways as gerotherapeutic targets, and we explore interventions like IH, HBOT, and IHHT not merely as “oxygen supplements,” but as molecular tools designed to retune the homeostatic machinery whose physiological efficiency and precision decline with age. Targeting the oxygen cascade offers a high‐leverage strategy to decouple chronological age from biological decay, providing a robust roadmap to extend human healthspan.

## Author Contributions

Conceptualization: Stefano Donega, Kenneth W. Fishbein, Luigi Ferrucci. Supervision: Luigi Ferrucci, Kenneth W. Fishbein. Writing (original draft): Stefano Donega, Kenneth W. Fishbein, Luigi Ferrucci, Myriam Gorospe, Paolo Dominelli, Allison B. Herman, Rafael de Cabo. Revision: Writing (Finalization and Editing): Paolo Dominelli, Myriam Gorospe, Allison B. Herman, RDC, Luigi Ferrucci.

## Funding

This research was supported in part by the Intramural Research Program of the National Institutes of Health (NIH). The contributions of the NIH authors were made as part of their official duties as NIH federal employees, are in compliance with agency policy requirements, and are considered Works of the United States Government. However, the findings and conclusions presented in this paper are those of the authors and do not necessarily reflect the views of the NIH or the U.S. Department of Health and Human Services.

## Disclosure

Figures were conceptualized and drafted by SD, structured using AI language developed by OpenAI, and finalized using Microsoft PowerPoint. The authors used OpenAI to improve the clarity and readability of parts of the manuscript and to generate the abbreviation list. The authors reviewed and edited the content and are fully responsible for the final version of the manuscript.

## Conflicts of Interest

The authors declare no conflicts of interest.

## Data Availability

The authors have nothing to report.
